# Patterns of tined lead migration in sacral nerve modulation

**DOI:** 10.1007/s00384-020-03530-0

**Published:** 2020-03-06

**Authors:** Emmanuel Ezra, A. M. Helene Siilin, Milan Gulobovic, J. Wilhelm R. Graf

**Affiliations:** 1 Department of Surgical Sciences, Uppsala University, Akademiska Sjukhuset, 75 185 Uppsala, Sweden; 2 Department Section of Radiology, Uppsala University, Akademiska Sjukhuset, 75 185 Uppsala, Sweden

**Keywords:** Tined lead, Migration, Sacral nerve modulation, Fecal incontinence

## Abstract

**Aim:**

Lead migration is a common cause of loss of efficacy in sacral nerve modulation. Our aim was to systematically study the migration pattern of tined leads in sacral nerve modulation. Our hypothesis was that tined leads may promote forward migration because of their configuration.

**Method:**

Consecutive patients treated with sacral nerve modulation with a tined lead electrode, who had experienced loss of efficacy and had radiographs both at baseline and after loss of efficacy between 2005 and 2016 were eligible for inclusion.

**Results:**

Twenty-five patients out of 70 with loss of efficacy were studied. Lead migration was measured as percent electrode movement in relation to sacral cortex at lateral projection. All had some degree of lead migration, ranging from 35% backward to 74% forward migration. Sixteen (64%) had forward migration while nine (36%) had backward migration. In seven patients (28%), loss of efficacy was associated with an episode of perceived mechanical strain on the electrode. Fifty percent (4/8) who associated their loss of efficacy with an adverse event had forward migration of the electrode.

**Conclusions:**

Forward lead migration with concomitant loss of efficacy seems to be a common event in patients with tined leads, hence supporting our hypothesis. The retrospective design and that some of the patients with loss of efficacy could not be included because of incomplete data, which is a limitation to the study. Further studies are needed to confirm to what extent the direction and magnitude of the migration relate to loss of efficacy.

**Trial registration:**

## Introduction

Sacral nerve modulation (SNM) is an established treatment for fecal incontinence (FI).

Loss of efficacy, defined as “reduced or ceased therapeutic benefit after a period of satisfactory results,” has been reported in up to 45% of cases, making it a major challenge in SNM [1].

Tined leads are constructed to prevent outward migration [2].

The association between lead migration and loss of efficacy has been addressed by several authors [3–7], but systematic studies of migration patterns are lacking.

The hypothesis of this study is that tined leads allow and may even promote inward migration, and the aim was to study lead movement in patients with loss of efficacy.

## Methods

### Materials

After institutional review board approval, all patients who had undergone a successful percutaneous nerve evaluation test (PNE test) for any reason (FI, constipation, and other indications) at the Department of Surgery, Uppsala University Hospital, between 2005 and 2016 were evaluated for inclusion through a review of relevant medical records. The inclusion criteria were:

1. Successful PNE test [8], i.e., a 50% reduction of incontinence episodes, alternatively, a 50% increase in number of bowel movements in patients with constipation.
2. SNM with a tined lead electrode.
3. Loss of efficacy with no improvement after changing the programming parameters.
4. Posteroanterior and lateral radiographs at baseline and after loss of efficacy.

**Fig. 1:**
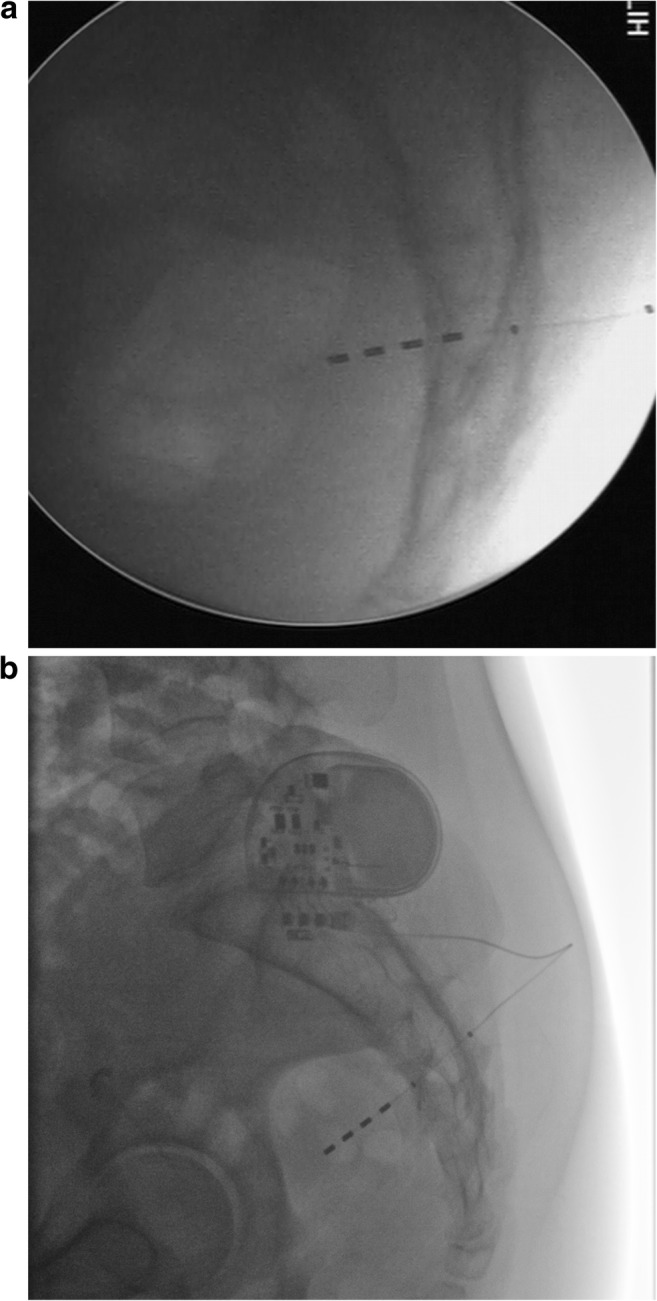
**a** Lateral pelvic radiography showing electrode position at implantation and **b** after loss of efficacy

The radiographs were assessed by a senior radiologist for lead disruption or migration. The distance between the lead tip and the ventral surface of the ventral sacral cortex where the lead exited the sacral foramina was assessed to determine whether migration had occurred and the direction of the migration (Fig. 1 a and b).

To avoid measurement errors because of variations in magnification, relative values of lead displacement were used in the calculations.

### Surgical technique and follow-up

During the first part of the study period, 2005 to 2011, the PNE test was performed using a thin, temporary electrode under general anesthesia without muscle relaxants with the patient in a jackknife position. After marking of bony landmarks verified with radiography, the third or fourth sacral foramina was punctured with a hollow needle and a temporary electrode inserted (Model 3057; Medtronic InterStim, Minneapolis, MN, USA*)*. The S3 nerve root was the preferred site of stimulation. The threshold of a perineal motor response was registered, and at least two temporary electrodes were implanted and fixed with an adhesive dressing. Postoperatively, the electrode with the lowest threshold causing a sensory response without discomfort was selected for initial stimulation and connected to an external stimulator (Interstim Model 3625; Medtronic, pulse amplitude 0.1–10 V; frequency 14 Hz; pulse with 210 ms) kept in the patient’s belt or pocket. From 2012 to 2016, the PNE test was performed with the tined lead electrode (InterStim model 3889). The procedure was done with local anesthesia and sedation, with the patient in a jackknife position, allowing location of the target sensory area, which is not possible during general anesthesia.

In cases of suboptimal treatment effect, the alternative test lead was used or the program parameters were changed. At the first follow-up visit, all temporary electrodes and tined lead electrodes with negative tests were extracted. Based on the diary registrations and patients’ interviews, a decision was made concerning a permanent implant, provided that a 50% symptom reduction had been achieved. In patients with the temporary electrodes with positive tests, the permanent implant was performed in a similar manner except that a permanent tined lead electrode (InterStim model 3093/3889) was used which was connected to the stimulator (InterStim model 3023) placed in the deep subcutaneous gluteal pocket through a connecting cable (InterStim model 3095). In patients with tined lead electrodes and positive tests, the implanted electrode was connected to the stimulator in the same manner. The stimulator was activated the day after the surgery. The patients also received a remote control and were instructed about the system and how to vary the amplitude and stimulation sites. Patients were instructed to avoid physical exercise, especially lumbar flexion or other excessive ranges of motion affecting the electrode, for at least 3 months post-implant, and a 4–6-week sick leave was recommended.

### Statistical method

Data are presented as means ± SD or medians and range. Lead migration was assessed by comparing lateral pelvic radiographs after loss of efficacy with baseline radiographs, using the Wilcoxon matched pairs test. *P* < 0.05 was considered statistically significant.

## Results

### Clinical course

In total, 136 patients had successful SNM and a permanent implant. Of these, 70 patients (52%) experienced loss of efficacy. Twenty-five out of 70 (36%) had complete radiographs before and after loss of efficacy and could be included for evaluation. Seventeen patients (68%) suffered from FI and eight (32%) from constipation. Ten patients (40%) were implanted in S3 dx, eleven (44%) in S3 sin, and four (16%) in S4 sin.

### Electrode migration

**Fig. 2:**
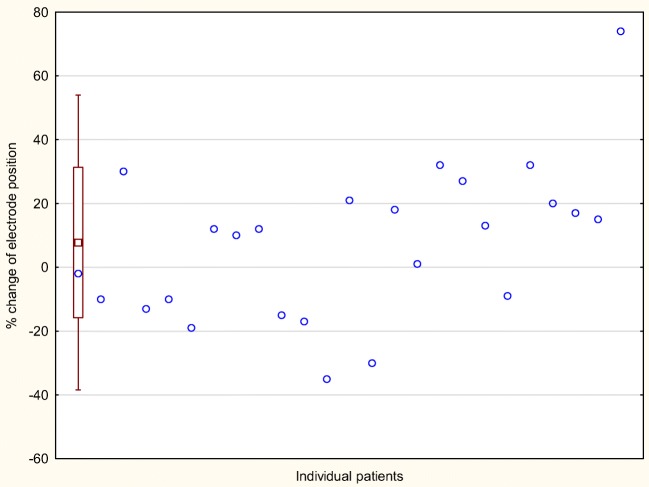
Individual and overall relative change of electrode position. Each circle represents the change of electrode position at follow-up–relative baseline. The box plot shows the mean change ± SD and the bars ± 1.96 × SD

Lead migration frequency in this population was 100%. The lead migration ranged from 35% backward to 74% forward, and the median migration was 12% (mean 8%) forward **(**Fig. 2**)**. The median percentage of lead extending beyond cortex was 62% at baseline and 71% after loss of efficacy (*P* = 0.14). Sixteen patients in the study population (64%) had forward migration, while nine (36%) had backward migration. Loss of efficacy was associated with an adverse event, i.e., mechanical strain, in seven patients (28%) and one patient (4%) experienced loss of efficacy in connection with a botulinum toxin injection for an anal fissure. In 50% (*n* = 4) of the patients who associated their loss of efficacy with an adverse event, a forward dislocation of the electrode was observed.

## Discussion and conclusion

Although SNM is used successfully for fecal incontinence, continuous improvements of implants, operative techniques, and minimization of side effects are all still needed. All these aspects were addressed in this study [9].

Of those who received SNM with tined leads, 92% had a positive result, in line with previously reported positive predictive values of a positive PNE test [10]. However, in our material (70 patients), 52% experienced loss of efficacy, which is slightly higher than the frequencies up to 45% reported by Maeda et al. [1].

Theoretically, differences in patient selection, surgical technique, and the size of this material may explain variations in the proportion of loss of efficacy.

All with repeated pelvic radiographs exhibited some degree of lead migration, and this is a significantly higher percentage than reported by others, e.g., Jarret et al. and Spinelli et al. [4, 5]. However, these authors also refer to small patient populations receiving SNM with tined leads and measurements of lead migration at 6 weeks and 6 months post-implantation respectively.

In clinical practice, it is believed that post-implantation electrode dislocation occurs early due to lack of scar fixation. Most centers give their patients physical instructions to avoid early dislocations. However, in our material, the mean duration from implantation to the radiograph indicated by loss of efficacy was 22 months. Although the dislocation might have started earlier, the symptom change occurred within a limited time frame before imaging. It is possible that dislocation is gradual and that loss off efficacy occurs at a specific point during this continued dislocation. Our findings indicate that early dislocation is probably not the major reason for loss of efficacy in this material. Nevertheless, since we do not have repeated radiographs before loss of efficacy has occurred, we cannot say anything about the dynamics of the dislocation.

In 50% (*n* = 4) of the patients who associated their loss of efficacy with an adverse event, a forward dislocation of the electrode was observed contrary to what could have been expected.

We used a standardized patient and c-arm setups in order to minimize variations in angulation and intra- and inter-patient variations in measurements. Still, despite our standardized setup and the use of relative values, possible measurement errors need to be considered in the overall interpretation. However, we believe that our methods are reasonably accurate in detecting the gross direction of movement in the electrode. The retrospective design and the patients lost to follow-up because of incomplete data are further limitations to this study.

Small movements of an implanted electrode are probably inevitable. The trend towards a predominately inward dislocation might suggest that tines facilitate inward micro movements making these electrodes more susceptible to inward dislocation. Deng et al. found a similar trend in 2006 [11]. A possible solution to this problem is the construction of leads with tines that prevent bi-directional migration.

Although considered cost-effective, SNM is a relatively costly treatment. Lead-related problems account for almost two-thirds (58.8%) of unplanned surgical procedures after implantation [12]. Therefore, minimizing loss of efficacy due to technical failure, for example lead migration, is important.

In this material, forward lead migration with concomitant loss of efficacy seems to be a frequent event in patients with tined leads, hence supporting our hypothesis. To confirm if and to what extent the direction and magnitude of the migration relate to loss of efficacy needs further studies.
